# On Some Postepileptic Phenomena

**Published:** 1883-10

**Authors:** 


ON SOME POSTEPILEPTIC PHENOMENA.

In a paper read in the Section of Medicine, at the Annual Meeting of the British Medical Association at Liverpool, August, 1883, Julius Althaus, M.D., M R.C.P. Lond., Senior Physician to the Hospital for Epilepsy and Paralysis, Regent's Park, makes the following introductory observations before describing a series of cases: "I wish to draw attention to certain either acute or chronic alterations of the mental faculties which have fallen under my notice, as direct consequences of epileptic attacks. I shall purposely exclude, in discussing this matter, any cases in which epileptiform seizures took place in consequence of gross organic lesions, such as tumour of the brain, chronic inflammation of the membranes and the gray surface of that organ, blood-poisoning of various kinds, and other diseases in which the convulsive paroxysms were only one symptom amongst many others; and I shall confine myself strictly to the consideration of those cases in which epilepsy occurred as a true neurosis, that still mysterious and unexplained functional disease of the gray matter of the brain, which is possibly owing to some kind of imperfect nutrition, but certainly not to any such structural alterations as would reveal themselves to our present means of research.

"The paper is based on an analysis of the cases of 250 epileptic patients which have been under my care in private and hospital practice during a period of six years. Amongst these cases there were 89, or 35.6 per cent., in which no preceptible temporary or permanent alteration in the mental condition, which could be ascribed to the epilepsy, was to be ascertained ; while in 161 cases, or 64.4 per cent., such alterations did occur. Of the 89 cases which escaped mental deterioration, 61, or 68.5 per cent., were instances of nocturnal epilepsy, while in 28, or 31.4 per cent., attacks took place in the daytime. All, however, which escaped were cases of typical convulsive attacks ; while in all cases of loss of consciousness without convulsion, or *petit mal*, and epileptic vertigo or automatism, a more or less permanent mental alteration was induced. Among the 161 cases which were followed by mind-affection, there were :

123 cases (or 76.5 per cent.) of typical convulsive attacks ;
26 " (or 16.1 per cent.) of *petit mal ;* and
12 " (or 7.4 per cent.) of epileptic automatism.

"Among these patients there were 91 males, or 56.5 per cent., and 70 females, or 43.5 per cent. The ages of the whole series varied from 5 to 62 ; and when these were distributed over decades, it appeared that the decade from 5 to 15 was at the bottom of the list with 10.5 per cent.; while that between 15 and 25 headed the list with 24 per cent., the other decades being very nearly even, with a medium of about 16 per cent. The hereditary influence was marked in 66 cases, or 40.9 per cent. The nature of other predisposing or exciting causes, as far as they could be ascertained, did not appear to have exerted any special influence, since they were much of the same kind as in those cases in which the mind was not affected. I will, in passing, remark that I have excluded from the present considerations those cases which were apparently owing to injury to the head, syphilis, and masturbation, as these are of a complex character.

" In cases, therefore, which form the groundwork of this paper, are only such where epilepsy was the primary event, and where some mental disturbance was observed subsequently to, and as a direct consequence of, the attacks. There are two forms of this disturbance, viz.: an acute one, where mental symptoms occur soon after attacks, and disappear again after a certain time ; and a chronic form, in which there is a gradual and permanent loss of mental power consequent upon attacks.

"The characteristic feature of the acute form of postepileptic mental affection is its periodicity. Identical, or at least highly similar, symptoms are seen to occur year after year, and gradually become intensified, unless they be checked by active treatment. They do not always occur immediately after attacks, but occasionally a day or two afterwards, and last a variable time, but rarely longer than a week. After such an attack is over, the patient has mostly no recollection whatever of what has occurred."—*British Medical Journal.*

